# Establishing a skills laboratory in a Brazilian University Hospital: introducing clinical simulation for pediatric emergencies

**DOI:** 10.3389/fmed.2025.1579250

**Published:** 2025-11-05

**Authors:** Fernando Belluomini, Andrea de Melo Alexandre Fraga, Fernando Augusto Lima Marson, Lucas Silva Mello, Patrícia Teixeira Costa, Paulo Cesar Massucatto Colbachini, Angélica Maria Bicudo

**Affiliations:** ^1^Simulation Center, School of Medical Sciences, University of Campinas (Unicamp of the Portuguese Universiade de Campinas), Campinas, São Paulo, Brazil; ^2^Department of Pediatrics, School of Medical Sciences, University of Campinas (Unicamp of the Portuguese Universidade de Campinas), Campinas, São Paulo, Brazil; ^3^LunGuardian Research Group—Epidemiology of Respiratory and Infectious Diseases, São Francisco University (USF of the Portuguese Universidade São Francisco), Bragança Paulista, São Paulo, Brazil; ^4^Laboratory of Clinical Microbiology and Genetics, São Francisco University (USF of the Portuguese Universidade São Francisco), Bragança Paulista, São Paulo, Brazil; ^5^Laboratory of Molecular Biology and Genetics, São Francisco University (USF of the Portuguese Universidade São Francisco), Bragança Paulista, São Paulo, Brazil; ^6^Pediatrics, School of Medicine, Pontifical Catholic University of Campinas (PUC-Campinas of the Portuguese Pontifícia Universidade Católica de Campinas), Campinas, São Paulo, Brazil

**Keywords:** clinical training, emergency pediatrics, medical residency, medical simulation, pediatrics, teaching

## Abstract

**Background:**

The history of Emergency Pediatrics at the University of Campinas (Unicamp of the Portuguese *Universidade de Campinas*) began in 1986, with the creation of the Discipline of Emergency Pediatrics in the Department of Pediatrics. This development was driven by the growing need for specialization in childcare, given the physiological differences and the specific attention required in emergencies. In the 1980s, the Pediatric Advanced Life Support (PALS) program played a crucial role in improving pediatric emergency care. Training in the area was formally regulated in 2016, after a period of interruption in the 2000s.

**Methods:**

This informative article was designed with the objective of underscoring the importance of establishing a skills laboratory within a university hospital center. To achieve this, we reviewed institutional records, the history of residency programs, and national and international initiatives that supported the inclusion of simulation in medical education. Special attention was given to the evolution of medical simulation, its integration into the Unicamp curriculum, and its application in pediatric emergency training.

**Results:**

Unicamp pioneered the creating of a residency program in Pediatric Emergency, consolidating its training with internships in Intensive Care Units and Anesthesiology. Since 1986, the Pediatric Emergency Room has been coordinated by institutional professionals and was strengthened with the start of the residency program in 1991. Training expanded with the creation of an outpatient clinic for graduates, and in 2018, 24 professionals were awarded the title of Pediatric Emergency Physician, three of them from Unicamp. Currently, 12 graduates are working in different regions of Brazil. Regarding simulation, Brazil began using this educational technique in the 1990s, initially in nursing courses. Following the World Health Organization’s recognition in 2009 of its importance in controlled training environments, and subsequent initiatives by the Ministry of Health and the Pan American Health Organization in 2014, medical simulation was progressively incorporated into Brazilian medical schools. At Unicamp, the implementation began in 2007 with the construction of the Center for Realistic Simulation, inaugurated in 2009. Since then, medical students and pediatric residents have participated in high-fidelity simulation sessions, strengthening both technical and non-technical skills such as communication and teamwork.

**Conclusion:**

The experience of Unicamp demonstrates that the establishment of a skills laboratory within a university hospital contributes significantly to the advancement of medical education, the consolidation of pediatric emergency residency training, and the improvement of healthcare delivery. Medical simulation, fully integrated into the curriculum, has proven to be an essential tool not only for developing clinical competencies but also for fostering professional collaboration, thereby strengthening the overall quality of pediatric emergency care.

## 1 Introduction

Contemporary medical education demands more than theoretical knowledge: it requires practical skills, refined clinical reasoning, and interpersonal competencies that ensure patient safety and quality of care. In this context, clinical simulation emerges as an innovative and effective pedagogical strategy, capable of transforming traditional teaching into realistic and safe experiences. This article discusses the relevance of implementing a skills laboratory in a university hospital setting, with a focus on pediatric emergency care, highlighting how such a facility can address historical gaps in medical training, promote teaching excellence, and stimulate applied research. The proposal is situated within a framework of curricular evolution, aligned with the National Curriculum Guidelines and the growing demand for professionals prepared to act competently, empathetically, and efficiently in critical situations.

## 2 History of the pediatrics emergency at the University of Campinas (Unicamp)

The Discipline of Emergency Pediatrics of the Department of Pediatrics of the Faculty of Medical Sciences was founded in 1986, 7 years after the consolidation of Emergency Medicine as a medical specialty in the United States of America (USA). There was a need for a vision directed at this age group, due to its physiological particularities, requiring differentiated treatment, with specific, trained and qualified staff, as well as proper and adequate equipment for the care of seriously ill children (1).

The standardization of care for children began to take shape with the unification of local and regional services into multidisciplinary care centers called Emergency Medical Services for Children. The idea was to reduce dysfunction, prevent death, and promote rehabilitation by systematizing the care of children and adolescents who were victims of potentially serious illnesses and traumas (2–4).

At the end of the 1980s, in order to disseminate knowledge of these care practices in the pediatric scenario, Pediatric Emergency training programs emerged, with the most popular being Pediatric Advanced Life Support, certified by the American Heart Association and aimed at medical professionals and nurses (5, 6). Although in the USA the training of these professionals became regular and frequent from the 1980s onwards, it was regulated and certified as a subspecialty in 1991 (7).

In Brazil, in the 1990s, the Brazilian Society of Pediatrics began a movement to stimulate and regulate training in various areas of pediatrics. In this context, the National Medical Residency Commission (NMRC) accredited some residency programs to offer an optional 3rd year of the Pediatrics Residency Program for training in a specific area (Emergency), which lasted until 2002. In that year, the specialties and areas of practice defined by the Brazilian Medical Association, the NMRC and the Federal Council of Medicine were unified, and Urgency and Emergency became exclusive areas of clinical medicine (8). As a result, the training of pediatricians with training and qualification in the field of emergency medicine was suspended for more than a decade. The hiatus in the training of qualified professionals in Emergency Pediatrics has been detrimental to the population of Brazil, to Pediatrics, to Teaching, and to Research.

In mid-2010, most Pediatric Emergency Residency programs began to be developed over 3 years, with the definition of a minimum curriculum, as well as the skills and competencies to be achieved. In recent years, the subspecialty has been consolidated as a source of knowledge, teaching, and research. Aware of the educational and training loss in Pediatrics, in 2015, 20 renowned institutions from Brazil met with the NMRC and presented proposals for Adult Emergency Residency programs (3 years) and Area of Expertise in Pediatric Emergency (1 year), with detailed program content, as well as the skills and competencies to be acquired (9). The NMRC decided to approve the request for accreditation of 22 residency programs proposed by these entities. As a result of this move, the specialty and area of practice were recognized by the Scientific Council of the Brazilian Medical Association in 2015 and formally approved with the publication of Resolution No. 2,149/2016 of the Federal Council of Medicine (10). Before recognition, there were only 2 residency programs in Adult Emergency. Since then, 52 new programs have been started. Brazil now has 54 residency programs in 16 of the 27 federative units. As a result, in 2020, 192 professionals were certified as Emergency Physicians in Brazil (11). With regard to Emergency Pediatrics, Brazil currently has the same 22 accredited programs. Compared to the demand, there is still a need for more training centers for these professionals. Furthermore, the expectation is to expand and increase the length of residency from 1 to 2 years (12).

Among the pillars of the Emergency Pediatrics Residency Program, the following stand out:

(i) A model based on practice, with preceptorship actively involved in the teaching process. Less frequent situations should be experienced through simulation laboratories or other teaching methods, such as filming, role-playing, and others;
(ii) Training leaders to work in Emergency and Urgency services;
(iii) Short-term increase in the number of pediatricians trained in Emergency and Urgency;
(iv) Development of research in Pediatric Emergency.

Currently, training in Emergency Pediatrics has returned talented individuals committed to improving the care of critically ill children to the practice scenario. They exercise leadership in their respective services and promote qualification in care, research and teaching, as well as acting decisively in their management (12).

The Pediatrics Emergency Room at the Clinical Hospital (*Hospital de Clínicas*) at Unicamp began operating in 1986 under the coordination of Dr. Rober Tufi Heten, with the participation of professors from the general outpatient clinic who dedicated part of their workload at the unit and rotated to cover their schedules. Dr. André Moreno Morcillo, Dr. Roberto Nakashima, Dr. Roberto Marini, and Dr. José Dirceu Ribeiro were some of the teachers who continued to perform shifts for a long time. As the number of patients evolved, two former students from the Faculty of Medical Sciences and graduates of the Pediatrics Residency were hired: Dr. Gil Guerra Jr. and Dr. Emilio Carlos Elias Baracat. Dr. Rober Tufi Heten remained head of the Emergency Room for a short time and Dr. Emílio Baracat took over in 1987. Subsequently, Dr. Mariana Porto and Dr. Roberto José Negrão Nogueira, who had specialized, respectively, in the General Outpatient Clinic and the Paediatric Intensive Care Unit and Ward, were hired, guaranteeing the coverage and quality of care and the teaching needs for students from the Faculty of Medical Sciences and resident physicians from the Paediatrics Department. In 1991, the idea of an Emergency Pediatrics Residency began, a subspecialty that was beginning to emerge abroad, especially in the USA. In this context, it was important to assess the most prevalent critical situations at the time and provide training in what was believed to be necessary for a doctor working in Emergency Pediatrics. Drs. Gil Guerra Jr. and Emilio Carlos Elias Baracat, together with Dr. Roberto José Negrão Nogueira, set up a grid of activities associated with the most prevalent situations in the Pediatric Emergency Room at that time and began to provide training in what was believed to be necessary for a physician working in the proposed area.

The candidate for the first vacancy was Dr. Marcelo Conrado Reis, who was initially trained in the vaccine reactions that were occurring at the time, accidents involving venomous animals, trauma, emergency procedures, and treatment of the most prevalent acute diseases, creating an outpatient clinic for observation graduates that was conducted by R3 (third-year residents), advised by Drs. Gil Guerra Jr. and Emilio Carlos Elias Baracat. Training was also conducted through participation in management meetings of the Emergency Services, which were in the structuring phase. Over the years, the program has been improved with the creation of internships at the Pediatric Intensive Care Unit, Anesthesiology, Poison Control Center, Mobile Emergency Care Service, among others, as well as the possibility of attending internships at other services in Brazil and abroad, such as Cincinatti Children’s Hospital in the USA.

In May 2018, during the II Brazilian Congress of Urgencies and Emergencies in Pediatrics, a select group of 24 professionals were awarded the title of Emergency Pediatrician for proficiency. Proudly, three of them (Marcelo Conrado dos Reis, Andrea de Melo Alexandre Fraga, and Fernando Belluomini) are still part of the team, as well as Emílio Baracat, who has already retired. The service currently has 12 graduates working in various regions of the state of São Paulo and Brazil, providing care, teaching, and managing pediatric emergencies. Graduates are also involved in the Pediatric Emergency Department of the Brazilian Society of Pediatrics and the São Paulo Society of Pediatrics, in management positions.

## 3 Historical milestones of medical simulation

### 3.1 International

Simulation is defined as an attempt to present authentic situations and problems, with the aim of teaching, training, and/or evaluating individuals, within a controlled and safe environment (13, 14). In healthcare, the set of conditions that make up a simulated scenario tries to represent the patient and their clinical conditions as authentically as possible, with a greater or lesser degree of fidelity and complexity, depending on the learning objectives (15, 16). The first initiatives regarding simulation derive from the practice of acupuncture in the 10th century. Feedback was carried out in real time, providing the anatomical location for the placement of the instruments on the respective structures (17, 18). Until the 20th century, healthcare simulation was limited to low-complexity training to demonstrate the acquisition of skills, particularly in the areas of obstetrics, general surgery, and ophthalmology (19). In this context, some moments in history mark the development of clinical simulation:

(a) 1940s: Asmund Lærdal, together with a Norwegian publisher and toy manufacturer, developed Resusci-Anne, a “task trainer” (or “skills trainer”) that would revolutionize resuscitation training because it was effective and inexpensive (19). Since then, evolution has been constant, with an increasingly sophisticated range of mannequins and the spread of models used to support resuscitation and basic skills training.
(b) The 1960s saw the development of more complex simulators dedicated to reproducing clinical and realistic aspects of the human being. The first prototype developed by Denson and Abrahamson (20) was the “Sim One.” The mannequin had sophisticated features such as breathing, heartbeat, temporal and carotid pulses (all synchronized), blood pressure; mouth opening and closing; eye blinking; response to four drugs administered intravenously and two gases (oxygen and nitrous oxide), administered through a mask or tube. The mannequin’s supposed physiological responses were in real time and programmed by a computer (15). Despite promising reports of their effectiveness in training, the cost was very high and the concept of teaching at the time did not include the use of innovative simulators.
(c) 1980s: the feasibility of producing high-fidelity simulators was revived by two groups, the first at Stanford University and the other at the University of Florida. The first group, led by David Gaba, developed the Comprehensive Anaesthesia Simulation Environment (CASE) and the second, led by Michael Good and Joachim Stefan Gravenstein, developed the Gainesville Anaesthesia Simulator (GAS) (15). The Stanford team focused on developing teamwork in realistic simulation environments. They incorporated a crisis management model used by aviation, with significant results. This type of training would become the embryo of the non-technical skills of teams (21). These simulators and some European counterparts (from Holland, Denmark, and the United Kingdom) formed the basis for today’s moderate to high fidelity simulators.
(d) The latter part of the century saw the beginning of the reform of medical education that persists to this day. With the recognition of the information overload in the undergraduate curriculum, to the detriment of learning clinical and communication skills, the adoption of programs covering clinical skills and the development of teaching facilities to support this learning began (22, 23). There was also a need for continuing medical education after specialized higher education, which led to another application for simulation in postgraduate studies. Another point raised in relation to simulation is that it is a safe, secure environment where students and postgraduates are free to make mistakes, discuss the reasons for the error, and train again as many times as necessary (24). Some publications have also highlighted patient safety and emphasized the need for an institutional approach to overcome individual and cultural barriers. Within this approach, the inclusion of simulation as a teaching method becomes a key tool (25, 26).

### 3.2 National

In Brazil, clinical simulation began with occasional activities in the early 1990s, mainly with advanced life support courses such as Advanced Trauma Life Support and Advanced Cardiovascular Life Support (27). At that time, there was no regular inclusion of simulation activities in the curriculum, training was restricted to skills and was adopted more by nursing courses than medical courses. As such, the use of clinical simulation as a form of teaching remained in Brazil in isolated activities carried out in a few locations, mainly in the southern region of the country (28). Similarly, the use of clinical simulation for Objective Structured Clinical Examination-type evaluation activities occurred in some medical courses, but only sparsely, with the personal efforts of teachers who overcame barriers, until this modality established itself as an excellent evaluation strategy (29).

In 2009, the World Health Organization defined the concept of Patient Safety as reducing unnecessary risks and harms associated with health care to an acceptable minimum. As a result, the traditional “see and do” approach in teaching medicine, exposing patients to the care of inexperienced health professionals, was no longer sustainable from an ethical point of view. Simulation, in this context, has emerged as an instrument that helps in the training of health professionals in skills training and simulated scenarios, anticipating clinical practice, without exposing patients to avoidable errors due to a lack of adequate knowledge and safety in carrying out procedures (30).

As part of the changes to the National Curriculum Guidelines (NCGs) for Undergraduate Medicine Courses in 2014, the Ministry of Health and the Pan American Health Organization established a partnership with the *Escuela Andaluza de Salud Publica*, which dedicated a specific component to learning through simulation—an important component to be developed in Brazil. At that time, it was up to the health and educational authorities to promote and disseminate the method in learning processes, through the articulation and integration between teaching and service. The partnership with *Empresa Brasileira de Serviços Hospitalares* (Brazilian Hospital Services Company) was of the utmost importance as it was starting to structure simulation centers in its hospitals. The link with medical schools, through the Brazilian Association of Medical Education, made it possible to identify professionals who were specializing in this area. In 2014, the 1st Training Workshop on Competencies and Simulation was held at the Federal Universidade de São Carlos (University of São Carlos)/SP, Brazil, with the team from the IAVANTE Institute of Andalusia, whose objective was to define a training model based on simulation, valuing the improvement of care processes in order to, at the same time, meet the training needs of professionals from the different states of Brazil (31).

Another milestone took place at the Brazilian Congress of Medical Education in 2015, in Rio de Janeiro city, Rio de Janeiro (RJ), where there was a practical skills and simulation program for students and a theoretical program designed for teachers. At the following congress, the simulation program evolved into the demonstration of scenarios in Brasília, Federal District (FD) (2016) and culminated with the Simulation Olympics in Porto Alegre, Rio Grande do Sul (RS) (2017) and Vitória, Espírito Santo (ES) (2018). At the 2019 competition in Belém, Pará (PA), there were three simultaneous rooms using highly complex mannequins, clinical cases from Body Interact and scenic simulation, allowing interaction between the participating teams and discussion of the clinical cases with the audience (31). Currently, following a partnership between the Brazilian Association of Medical Education, the Brazilian Network of Hospital Services of Federal University Hospitals, and the Pan American Health Organization, a course is being developed to train and educate simulation trainers (32). In this context, future projects are being directed towards offering the course to associated medical schools and setting up the Simulation Centers of the Regional Offices of the Brazilian Association of Medical Education. The objective is to develop the learning method in geographical districts of operation and to interact with medical schools and their members, and to create a collaborative network in view of the diversity of contexts and situations that exist in terms of the presence of skills and simulation laboratories and the use of clinical simulation in the curricula of medical courses (31). The organization will be an important step for the development of simulation in Brazil, enabling the creation of a collaborative network of centers and teachers (33).

## 4 Insertion of clinical simulation in the teaching and learning process

Even today, many health courses have their curriculum organized into subjects. In this traditional structure, students are first introduced to theory and then have contact with practical situations. This model implies that students will be able to apply what they have been taught naturally and appropriately, moving from the abstract to the concrete (34). In practice, this training model results in a gap between the profile of the professionals who are entering the job market and the real health needs of users. Therefore, many medical schools have already moved towards other models, albeit partially, seeking greater interdisciplinarity and early application of knowledge (35). This has led the world to undergo changes in educational environments. The development of technologies and new teaching dynamics are the main contributors to this transformation. An important fact to remember that decisively contributed to intensifying these changes was the coronavirus disease 2019 (COVID-19) pandemic, which challenged the world to find new and stimulating tools for teaching (36–39). In this context, the use of simulation as a learning tool has proven to be effective (33).

The first reports of the use of simulation as a teaching and learning tool date back centuries ago, in biblical records; it has also been well described in the aviation and aerospace industries, in the army, in commercial airlines, and in nuclear power plants. In the health sector, there are records dating back to the early 17th century, with models used in anatomy, obstetrics, anesthesia, and neurology (40–42). As it is an educational strategy that uses structured activities, where conditions are created or replicated to represent real or hypothetical situations in a simulated environment, clinical simulation gives students the opportunity to experience situations of errors and successes, systematically report and discuss these errors and near accidents, recognize unsafe conditions, investigate, repeat procedures until they are correct, improve systems with a complete understanding of human fallibility and train in communicating errors to patients and families (43–45).

Over the last two decades, simulation-based teaching has been on the rise and has now become a component of health education at undergraduate, postgraduate, continuing and permanent education levels. As a result of the new needs in the health sector, there are increasing demands for more creative and problem-solving professionals. In this context, the teaching-learning process has evolved into a perspective of building knowledge in which teachers and students actively participate. The adoption of active methods to encourage effective student participation in the learning process has been growing, especially with the use of simulation (46). Simulation as a teaching and learning strategy in health education is complex because, although there is evidence that simulation can be effective, this depends on how it is practiced since it needs to be formulated appropriately so that it actually increases knowledge and improves professional and clinical skills and behaviors (43, 47). Therefore, if there is an intention to work with simulation in educational practice, it is essential to understand it as a teaching and learning method, because the use of simulated training in healthcare without an adequate theoretical basis does not ensure efficient results.

The theoretical foundations that guide the practice of simulation are associated with the concept of competence-based teaching. The development of this concept was enhanced with the establishment of competency milestones and, subsequently, with the development of Entrustable Professional Activities (EPAs). The concept of competence was developed throughout history, culture, and societal development and it was first described in a structured way by McClelland (44), focusing on the areas of teaching and administration. The concept of competence has three distinct pillars: knowledge, skills, and attitudes. These three dimensions are combined with attributes that involve cognitive, technical, social, and affective aspects for the success of a work activity (48).

**Figure 1:**
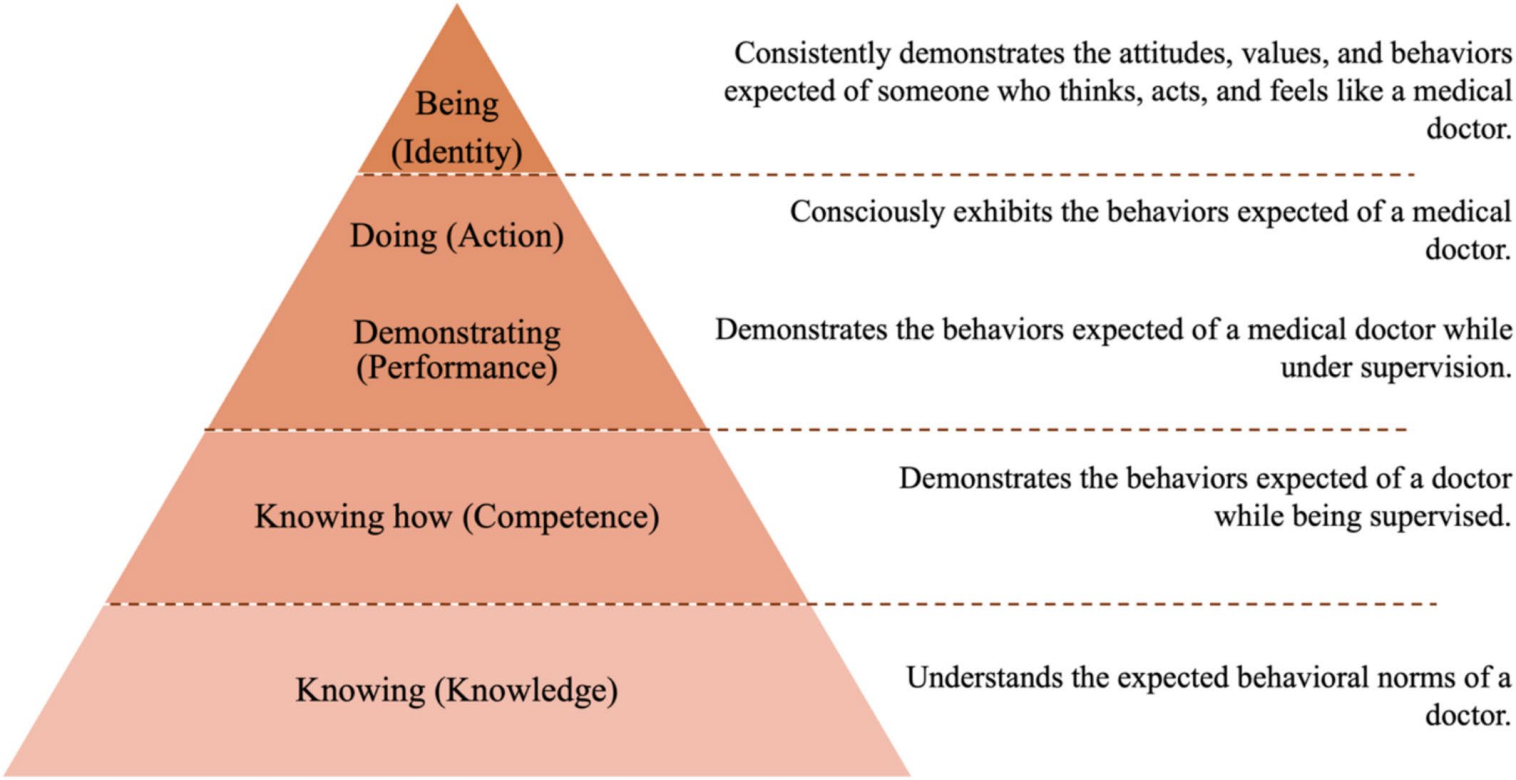
The corrected version of Miller’s pyramid, with the addition of “being” and an outline of what should be evaluated at each level.

In the health sector, the most widespread learning model is that of Miller (46)—It includes different levels of training that support professional competence and can determine its evolution. It is geometrically represented by a pyramid containing: (base) the knowledge required for effective professional performance; (second level) the subject’s ability to use their knowledge; (third level) representing how they demonstrate their skills in a given situation; and (peak) their action in real clinical situations (49). Currently, studies have discussed adding another level of assessment to Miller’s pyramid (46). This new level is above “doing,” which is the “being” level, in which professional identity, values, behaviors, actions, and professional aspirations are now considered (50, 51) (Figure 1).

The 2014 NCGs state that competence “is understood as the ability to mobilize knowledge, skills, and attitudes, using the available resources; it is also the ability to take initiatives and actions that translate into performances capable of solving, with relevance, opportunity, and success, the challenges that arise in professional practice in different contexts of health work, translating the excellence of medical practice” (52). The role of a competency matrix is to express the collective consensus about what is essential, and the interrelationships between the essential content that no student should fail to know when they graduate (52, 53). To this end, the NCGs recommend that medical training should be based on competences, and teaching by competences implies developing the students’ ability to mobilize knowledge, skills, and attitudes to deal with real-life situations, problems, and dilemmas.

The NCGs are essentially aimed at making the curriculum more flexible by aiming for a solid education in accordance with the knowledge developed in each area, but allowing students to contextualize the changes in the health area and their impact on the world of work, stimulating a generalist professional profile that promotes comprehensive, technical, scientific, humanistic, critical, and reflective care, prepared to work at the different levels of health care (54). The NCGs set out the principles, foundations and aims of medical training in an organized way, which is in line with the current situation in which medical schools must provide society with professionals who have a broad vision, are aware of their social relevance and are trained ethically, technically, and scientifically. The proposals and challenges encountered in implementing the new NCGs are widely discussed. Decentralizing teaching from the teacher and shifting it to the student, as the active subject of their training process, is an arduous task, since we have to break with the fragmentation of the disciplinary teaching model and build an integrated model whose training axis becomes practice, work, and care. The discussions aim to integrate theory and practice, changing the current model in which theory is presented in a disconnected way, preceding practice. As such, the establishment of the NCGs has led to changes in the curricular structure of various courses and in the projects of health education institutions (52).

The competency milestones, which can be seen as an evolution of competencies, describe in narrative form the competencies that develop over a period of professional training, and which must be demonstrated throughout the student’s period in clinical environments of different levels of complexity. They are organized in such a way as to show the results of the progressive development of students based on competences, from the time they enter university to postgraduate studies. In this way, they provide an overview and progression to assess the development of the medical student, also reaching the doctor in a specialty or subspecialty and, subsequently, the recertification of the specialist physician (55, 56).

The competency milestones are descriptions of the knowledge, skills, and attitudes for each of the competencies expected during the student’s training. This allows for the possibility of formative feedback to encourage changes in observed behavior. This model has spread across medical specialties such as General Surgery and Urology, with an emphasis on Emergency Medicine, whose advances in multi-professional care are taking place all over the world (49, 57, 58). Numerous competency milestones were created, but they lacked integration, synthesis and applicability. In this context, greater specificity was needed to assess apprentices in practical activities. Thus, the concept of EPAs was conceived—units of professional practice aligned with clinical care, which translate the elements of clinical practice into levels of proficiency. This is a way of defining the competences needed for graduates to be able to carry out their professional activity reliably. In this context, with the applicability of competency milestones, combined with the use of EPAs in the assessment process, clinical simulation fits in as an active teaching method (59).

The principle of simulation-based education is the transfer of competencies acquired in simulated activities, in which training allows for the acquisition of cognitive, affective, and psychomotor skills acquired outside the workplace and can serve to improve qualification and safety in solving patient problems in the clinical environment, with the realism of simulated teaching environments being a possible factor influencing the transfer of cognitive, affective, and psychomotor learning to the workplace. Pre-training on the simulator or in the simulated environment before meeting the patient addresses issues of safety in conduct and procedures, thus protecting institutions from ethical problems (60). However, simulation-based medical education is a complement to and does not replace existing educational methods and strategies in the traditional clinical environment to ensure that trainees become competent. After the simulation-based learning experience to develop the minimum level of competencies for providing safe care, students need to practice under supervision on real patients (61).

In order to develop and assess competencies in simulated activities, competency milestones have been recommended and are used to assess student progression. It is known that EPAs were originally created to be developed in the context of practice, however there are studies in the medical field that demonstrate the possibility of them being carried out in clinical simulation, due to the similarity of this method with the real context of clinical practice in health. EPAs can provide a platform for confident decisions around key skills, thus bridging the gap between theory and practice (62).

There are many practical challenges that need to be overcome when implementing simulation training programs, such as the short time determined for the activity, the lack of training for teaching staff, the high cost of the equipment and its maintenance, as well as restrictions on the space needed to set up activity laboratories. As simulators are no substitute for educators trained in good educational principles and teaching techniques, these teachers need to be identified and receive ongoing training in the use of simulation (60). To summarize, the use of competency milestones allows students to have an overview of their learning roadmap and identify the gaps in their training in order to achieve the essential competencies. Ultimately, these milestones give students a comprehensive overview of their learning, and they provide teachers with a valuable instructional guide.

The EPAs allow supervisors to systematize the assessment of student’s competencies, which must be reliably demonstrated when carrying out practical activities. Because they are objective, synthetic, and integrated, they ensure quality in instruction and evaluation and contribute to patient safety. In this way, it is possible to apply competency milestones and EPAs in healthcare courses, as they complement each other in the process of teaching and assessing competencies, and can be used to assess them in simulated activities. Incorporating these concepts and their practice into updated curricular matrices is currently a mandatory requirement for health courses, particularly in medicine.

## 5 National Curriculum Guidelines for the undergraduate course in medicine

### 5.1 Profile of the graduate/professional

A physician with a generalist, humanist, critical, and reflective education. Qualified to act, based on ethical principles, in the health-disease process at its different levels of care, with health promotion, prevention, recovery, and rehabilitation actions, from the perspective of comprehensive care, with a sense of social responsibility and commitment to citizenship, as a promoter of the integral health of the human being.

### 5.2 Competencies and skills

General knowledge, skills, and abilities:

- *Health care*: Health professionals, within their professional scope, must be able to carry out health prevention, promotion, protection, and rehabilitation actions, both on an individual and collective level. Each professional must ensure that their practice is carried out in an integrated and continuous manner with the other levels of the health system. Professionals must carry out their services to the highest standards of quality and in accordance with the principles of ethics/bioethics, bearing in mind that the responsibility for health care does not end with the technical act, but with the resolution of the health problem, both at an individual and collective level;
- *Decision-making*: The work of health professionals must be based on the ability to make decisions aimed at the appropriate use, effectiveness and cost-effectiveness of the workforce, medicines, equipment, procedures, and practices. To this end, they must have the skills to evaluate, systematize and decide on the most appropriate course of action;
- *Communication*: Health professionals must be accessible and must maintain the confidentiality of the information entrusted to them when interacting with other health professionals and the public. Communication involves verbal and non-verbal communication and writing and reading skills; mastery of at least one foreign language and of communication and information technologies;
- *Leadership*: In multi-professional teamwork, health professionals must be able to take on leadership roles, always with the well-being of the community in mind. Leadership involves commitment, responsibility, empathy, decision-making skills, communication, and effective and efficient management;
- *Administration and management*: Professionals must be able to manage and administer the workforce, physical and material resources and information, in the same way that they must be able to be managers, employers or leaders in the health team;
- *Continuing education*: Professionals must be able to learn continuously, both in their training and in their practice. In this way, health professionals must learn how to learn and have responsibility and commitment to the education and training of future generations of professionals, not just by transmitting knowledge, but by providing the conditions for mutual benefit between future professionals and service professionals.

Specific knowledge, skills, and abilities:

- Promote healthy lifestyles, reconciling the needs of both their patients and their community, acting as an agent of social transformation;
- Act at the different levels of health care, with an emphasis on primary and secondary care;
- Communicate properly with co-workers, patients, and their families;
- Inform and educate patients, family members, and the community about health promotion, disease prevention, treatment, and rehabilitation, using appropriate communication techniques;
- Proficiently carry out anamnesis and master the art and technique of physical examination;
- Master basic scientific knowledge of the bio-psycho-socio-environmental nature underlying medical practice and have critical thinking when interpreting data, identifying the nature of medical practice problems, and solving them;
- Correctly diagnose and treat the main diseases of the human being at all stages of the biological cycle, taking as criteria the prevalence and morbid potential of the diseases, as well as the effectiveness of medical action;
- Recognize their limitations and appropriately refer patients with problems that fall outside the scope of their general training;
- Optimize the use of propaedeutic resources, valuing the clinical method in all its aspects;
- Practice medicine using diagnostic and therapeutic procedures based on scientific evidence;
- Use semiologic examination and therapeutic resources that are scientifically validated, contemporary, and hierarchical for comprehensive health care at the first, second, and third levels of care;
- Recognize health as a right and act in such a way as to guarantee comprehensive care, understood as an articulated and continuous set of preventive and curative actions and services, both individual and collective, required for each case at all levels of the system’s complexity;
- Act to protect and promote health and prevent disease, treat and rehabilitate health problems, and monitor the death process;
- Perform clinical and surgical procedures that are essential for outpatient care and for the initial treatment of emergencies at all stages of the biological cycle;
- Know the principles of scientific methodology, enabling you to read technical-scientific articles critically and participate in the production of knowledge;
- Deal critically with labor market dynamics and health policies;
- Act within the hierarchical health system, obeying the technical and ethical principles of referral and counter-referral;
- Take care of their own physical and mental health and seek their own well-being as a citizen and as a physician;
- Consider the cost–benefit ratio in medical decisions, taking into account the real needs of the population;
- Have a vision of the social role of the physician and a willingness to take part in health policy and planning activities;
- Work as part of a multi-professional team;
- Be up to date with relevant health legislation. Based on these competences, the physician’s training should take into account the current health system in the country, the comprehensive health care in a regionalized and hierarchical system of reference and counter-reference, and the teamwork.

### 5.3 Curricular content

The essential contents of the undergraduate course in Medicine must be closely related to the most frequent health needs reported by the community and identified by the health sector. They should include:

- Knowledge of the molecular and cellular bases of normal and altered processes, of the structure and function of tissues, organs, systems and apparatus, applied to the problems of their practice and in the way the physician uses it;
- Understanding of the social, cultural, behavioral, psychological, ecological, ethical, and legal determinants of the health-disease process at the individual and collective levels;
- Approach to the health-disease process of the individual and the population, in its multiple aspects of determination, occurrence, and intervention;
- Understanding and mastery of medical propaedeutics—ability to perform clinical history, physical examination, and pathophysiological knowledge of signs and symptoms;
- Reflective capacity and ethical, psychological and humanistic understanding of the physician-patient relationship;
- Diagnosis, prognosis, and therapeutic management of diseases that affect human beings at all stages of the biological cycle, considering the criteria of prevalence, lethality, prevention potential, and educational importance;
- Health promotion and understanding of the physiological processes of human beings—pregnancy, birth, growth and development, aging, physical activities, sporting activities, and activities related to the social and environmental scene.

### 5.4 Course organization

The undergraduate course in Medicine must have a pedagogical project, built collectively, centered on the student as the subject of learning and supported by the teacher as the facilitator and mediator of the teaching-learning process. This pedagogical project should seek to provide the student with an integral and adequate education through an articulation between teaching, research, and extension/assistance.

The Curricular Guidelines and the Pedagogical Project should guide the curriculum of the undergraduate course in Medicine towards an academic and professional profile for graduates. This curriculum should also contribute to the understanding, interpretation, preservation, reinforcement, promotion, and dissemination of national and regional, international, and historical cultures, in a context of cultural pluralism and diversity. It may include complementary aspects of profile, skills, competencies, and content in order to consider the institutional insertion of the course, the individual flexibility of studies and the requirements, demands, and expectations of the development of the health sector in the region. The organization of the course must be defined by the respective course collegiate body, which will indicate the modality: annual series, semester series, credit system, or modular.

The structure of the undergraduate course in Medicine should:

o Have as the axis of curricular development the health needs of individuals and populations referred by the user and identified by the health sector;
o Use methods that favor active student participation in the construction of knowledge and integration between contents, as well as stimulating interaction between teaching, research, and extension/assistance;
o Include ethical and humanistic dimensions, developing attitudes and values oriented towards citizenship in the student;
o Promote integration and interdisciplinarity in coherence with the axis of curricular development, seeking to integrate the biological, psychological, social, and environmental dimensions;
o Introduce students early on to practical activities relevant to their future professional life;
o Use teaching-learning scenarios allowing the student to know and experience a variety of life situations, the organization of practice and multi-professional teamwork;
o Provide the active interaction of the student with users and health professionals from the beginning of their training, allowing the student to deal with real problems, taking on increasing responsibilities as a care and attention provider, compatible with their degree of autonomy, which is consolidated in the undergraduate course with the boarding school;
o Through teaching-service integration, link physician-academic training to social health needs, with an emphasis on the Unified Health System.

### 5.5 Internships and complementary activities

o *Internships*: Medical training includes, as an integral stage of the degree, a mandatory curricular internship of in-service training in a boarding school regime, in own or contracted services, and under the direct supervision of teachers from the School/University, with a minimum duration of 2,700 h. The in-service training mandatory curricular internship includes essential aspects in the areas of Clinical Medicine, Surgery, Gynecology-Obstetrics, Pediatrics, and Public Health, and should include activities at the first, second, and third levels of care in each area. The College of the undergraduate course in medicine may authorize, for a maximum of 25% of the total workload established for this internship, supervised training outside the federative unit, preferably in the services of the Unified Health System, as well as in a partner institution that maintains residency programs accredited by the National Commission for Medical Residency and/or other postgraduate programs.
o *Complementary activities*: Activities should be developed throughout the undergraduate course in Medicine and higher education institutions should create mechanisms for taking advantage of the knowledge acquired by the student through independent studies and practices in person and/or at a distance. The following can be recognized: monitoring and internships, scientific initiation programs, extension programs, complementary studies, and courses taken in other related areas.

### 5.6 Monitoring and evaluation

The implementation and development of the medical curriculum guidelines must be monitored and permanently evaluated to provide the necessary adjustments for their contextualization and improvement. The student’s summative and formative evaluations should be based on competencies, skills, and curricular content. The undergraduate course in Medicine must utilize methods and criteria for monitoring and evaluating the teaching-learning process and the course itself, in line with the evaluation system defined by the Higher Education Institution to which it belongs.

## 6 Simulation techniques and materials and simulators available for medical simulation training

Simulators are technologies that reproduce devices, physical parts or representations of services (63). During their undergraduate studies in the health area, even if they do not have a specific name, students have carried out some activity characterized as “Simulation”: Applying injections to fruit, training in the use of intraosseous needle devices in chicken bones, training surgical stitches by tying cotton threads to the chairs, and countless other “techniques” where the imagination allowed. These simulations were generally part of informal teaching and were not validated and studied as teaching tools (31). The importance of simulation in the process of training professionals who, in addition to theoretical content, have an improved ability to work in groups, with ease of communication and problem-solving, is already well established. For the best results to be obtained, it is imperative that the scenarios be as accurate as possible. However, it is important to establish that the overall fidelity of the simulated scenarios is hardly the same as the mechanical fidelity of the mannequins. Traditionally, overall fidelity has been described as a sum of “mechanical fidelity,” “environmental fidelity,” and “psychological fidelity.” There are also two other important contributors which are “temporal fidelity,” which refers to the recreation of events along a timeline as they happen, allowing for realistic time intervals between interventions and outcomes during simulated scenarios, and “action fidelity,” which represents the tasks assigned to students as dictated by the scenarios. The aggregate of all these fidelities is called “perception fidelity.” In the context of preparing the scenario and organizing the dynamics, some peculiar terms and concepts are often used. These are listed below (64, 65):

o *Virtual reality environment*: Computer-based application environment, commonly associated with 3D technology, which allows the participant to look at and navigate in an apparently real world;
o *Avatar*: Representation of a physical object (including a human being) in a virtual environment. It is a computer-generated graphic representation of a participant in a virtual reality simulation or game;
o *Facilitator (teacher/instructor/tutor)*: Trained individual who provides simulation-based teaching support and guidance. The facilitator must have mastery of and experience in clinical activity, technologies, and communication involving simulation. Ideally, this professional should have specific training in this area and be constantly retraining with an experienced mentor;
o *Feedback*: Information or dialogue between participants and facilitator about performance, with the aim of improving understanding of concepts or resolving doubts;
o *Technical skill*: The skill required to perform a specific task, for example, inserting a chest tube or carrying out a physical examination;
o *Non-technical skills*: Cognitive, social, and personal skills that complement technical skills in the adequate and safe performance of tasks, with various taxonomies for non-technical skills. In healthcare, the most used behavioral markers are communication, leadership/teamwork, task management, and decision-making;
o *Life savers*: Manage unexpected events that may occur during the simulation and/or interventions made during the scenarios, enabling participants to complete the simulated activity—“scenario saver”;
o *Moulage*: “make-up.” Applying make-up or devices that suggest wounds or fractures to actors or simulators. Some substances can also suggest blood, vomiting, and other excrement. The idea is to create scenarios with greater potential for realism. Moulage thus supports the participants’ sensory perceptions.

### 6.1 Simulation techniques

Clinical simulation has become a central pedagogical approach in health education, allowing students to develop technical, cognitive, and behavioral competencies in a controlled and safe environment. Various simulation techniques have been described in the literature, ranging from psychomotor skills practice on basic simulators to complex scenarios involving standardized patients or advanced virtual technologies. Each simulation modality is selected according to the learning objectives, the level of complexity of the competence to be trained, and the educational context, enabling the development of specific skills as well as the integration of communication, clinical reasoning, and multidisciplinary teamwork (17, 66–73). In this session, the main simulation techniques currently used in healthcare education will be described.

1. *Clinical simulation for skills training*: Practicing psychomotor skills with predefined learning objectives. Skills training allows the same technique or procedure to be repeated several times, developing skills of a lower level of complexity. The choice of the simulator will depend on the skill to be achieved;
2. *Clinical simulation using technological simulators (mannequins)*: This is one of the most widely used forms of practical teaching strategy in healthcare. It can be carried out with simulators of various technologies, depending on the focus to be given to the simulated case. Clinical reasoning, decision-making, technical skills, and multi-professional teamwork are examples of competencies that can be fostered by simulation;
3. *Simulation-based mastery learning*: This educational approach involves learners practicing procedural skills on task trainers or simulators until they achieve a predefined level of competence. The method emphasizes deliberate practice, frequent feedback, and assessment against objective performance standards, ensuring that all participants reach a minimum required proficiency before progressing to clinical application. SBML has been increasingly adopted in healthcare education due to its effectiveness in enhancing technical skills and improving patient safety outcomes;
4. *Clinical simulation with a simulated patient (actor)*: An actor plays a character, previously agreed upon, by means of a clinical script. This modality allows for greater realism in the scenario, where it is possible to evaluate the relationship between the professional and the patient;
5. *Role-play*: A learning method in which students are invited to take on the role of other professionals, different from the course they are taking. This resource is interesting because it allows students to see the scenario from another perspective and put themselves “in the other person’s shoes.” This dynamic is advantageous in terms of teamwork;
6. *Hybrid simulation*: Combining more than one simulation modality in a single training session, for example, using an actor in a scenario that includes mechanical simulators. The strategy allows for skills and communication training at the same time, in a more realistic way;
7. *Deliberate practice in rapid cycles*: A form of simulation in which a clinical case is applied to a group of participants repeatedly until the greatest number of them achieve the proposed, assertive result. Once the scenario is successful, the degree of difficulty can be increased;
8. *Virtual simulation*: A technological innovation where the scenario is virtual and presented on computer screens or multimedia. The student also interacts with the scenarios virtually;
9. *In situ simulation*: A strategy that reaches the pinnacle of fidelity and is carried out in the place where the hypothetical scenario takes place (74);
10. *Telesimulation*: A new teaching modality that has established itself as a very valid tool, especially since the COVID-19 pandemic. Scenarios can be filmed and presented to students remotely and discussed online by everyone. This allows for a greater inclusion of students, as it can reach many locations at the same time.

### 6.2 Simulators

There is no consensus in the literature regarding the classification of simulators. However, a widely adopted classification helps in setting up a Simulation Centre and is divided as follows: partial task trainers, patient manikins or simulators, simulated or standardized patients, screen-based environments, and simulated equipment and environments. Simulators do not necessarily have to be physical; they can take the form of a software or even occur in the minds of the students involved. However, one of the most widely accepted, and which allows for a good understanding and differentiation between models, is that which concerns the fidelity of the devices. These would be low, medium, and high fidelity. This classification refers to the type of technology used and the number of resources available in the manikins. This does not mean that the scenarios proposed with each manikin follow the same classification. A medium or high complexity scenario can be carried out using a low fidelity simulator, for example, and vice versa (63, 68, 71).

Low-fidelity simulators do not have anatomical, physiological, or sensory responses, therefore they do not act with the students in scenarios. They are generally used to develop or train skills such as venipuncture, bag-valve-mask ventilation, and tracheal intubation. They are called Part-task trainer when the models are shaped like organs or somebody segment (74, 75).

Medium-fidelity simulators, in addition to adding the functions of low-fidelity simulators, allow for greater veracity in cases, as the manikins can present features such as respiratory and/or cardiac auscultation and palpable pulses. These manikins are connected to a portable device where various types of tracings and other information pertinent to the case can be displayed (74, 75).

Finally, high-fidelity simulators are those capable of creating very realistic scenarios, as they have all the functions of the simulators described above, as well as the functions of more elaborate ones. For this sophisticated material to work properly, there needs to be a place set aside just for this activity, with separate rooms for installing the manikins, a control room where the computers with the software will be located and another room for the discussion of what has been accomplished in the scenario. There is such a great interaction between the scenario and the students that the manikins suffer the consequences of the medications or procedures performed (74, 75).

## 7 How to promote effective teaching using simulation

Technological developments in medical education, identified through research in this area, shape and channel educational policies. Educational technology and politics come together to promote an education based on the effectiveness, efficiency, and morale of students and teachers, through the introduction of new teaching and assessment models (43). One example is simulation-based medical education (76). In the literature, some topics were identified in a way that, together, they promoted high quality teaching in Simulation, namely (74, 76–86):

### 7.1 Feedback

The most important and frequently cited variable for promoting effective learning with the use of simulation. There are two ways of promoting feedback: formative or summative. Most feedback or debriefings are formative because the main goal is to improve the student’s clinical performance, rather than making summative judgments. For debriefings to be effective, they must be diagnostic, the ambience must be created thinking about facilitating learning, leaders and the rest of the team must be attentive to working together, one must make sure that team members feel comfortable during questioning, one must record the conclusions made and the goals set during the debriefing to facilitate feedback for future discussions.

### 7.2 Curricular integration

In order to fully achieve its objectives, simulation must be integrated with other teaching modalities, such as lectures, discussion of texts, laboratories, and supervised clinical practice itself. This means that simulation education and evaluation events must be carefully planned, scheduled and carried out in the context of a broad physician curriculum. Despite being an extremely effective teaching strategy, simulation cannot replace patient care in real clinics. Problems such as inertia and organizational barriers can hinder the curricular integration of simulation. For example, scheduling practice at simulation centers is a common problem. The pressure of commitments to the clinic, too many shifts, ingrained habits and perceptions that simulation is less valuable than the clinic can sabotage scheduled training sessions, reduce practice time and, consequently, not reach the full potential expected from this teaching modality. There are still practical issues to be established about the best approach to integrating simulation into existing curricula and the impact of this introduction on teaching staff and administrative resources.

### 7.3 Evaluation and measurement of results

An evaluation that allows the best measurement of results is important for any educational strategy. Reliable data is the necessary basis for educators to make valid decisions, judgments, or inferences about students. The first and most common form of assessment is observational assessment of student performance. Despite their ubiquity, they are subject to many sources of bias (unreliability) unless they are conducted under controlled conditions. Another method is based on student responses. Questions can be multiple choice, therefore selected or constructed. The reliability of response-based assessment is generally higher than observational assessment. An innovative form of assessment is carried out using “haptic” sensors—simulators that capture and record the touch the student makes in terms of location and pressure on anatomical sites, but studies are still needed to guarantee the reliability of this model. Historical records and recent research show that the measurement of simulation results by students is one of the biggest challenges currently facing the field. The research progress of this outcome evaluation is necessary to advance medical education and the effectiveness of simulation.

### 7.4 Simulation fidelity

A fundamental principle of simulation is that the educational objectives should dictate the decisions on the acquisition and use of the materials. The effectiveness of simulation will depend on a match between the educational objectives and the tools available. Teaching skills in basic procedures such as suturing, intubation, and lumbar puncture can be accomplished using simple task trainers. These are devices that mimic parts or regions of the body. More complex clinical events, which require team training in response to a particular event, require more sophisticated medical simulators. These are realistic, full-body manikins that have computer-controlled physiological characteristics, responding to physical interventions such as chest compressions and the administration of drugs and drug interactions, recording clinical events in real time and simulating many other parameters. More sophisticated simulators are virtual reality simulators, used for training in complex surgeries or cardiology procedures that require extreme skill.

### 7.5 Acquisition and maintenance of technical skills

The acquisition of technical skills is the most frequent learning objective of simulation. A growing number of studies are being conducted to evaluate the maintenance or deterioration over time of skills acquired in simulation environments. The results are mixed. Studies suggest that the decline in the ability to perform a certain skill depends on the type of specific skill acquired, the degree to which the skill was learned, and the time elapsed between learning and evaluation.

### 7.6 Transfer to practice

The transfer of knowledge and skills acquired in simulation to clinical practice is the highest level of the Kirkpatrick hierarchy, a theory that is used to classify the training results of these programs. Studies on simulation have documented the transfer of training to patient care environments, from the better response to cardiopulmonary arrest care by residents who had trained in this scenario in simulated environments, to a lower occurrence of catheter infections or other complications during its passage by residents who had previously trained on manikins.

### 7.7 Team training

In the literature, it is described that “patient care is a team sport.” There is evidence that communication (a skill totally dependent on teamwork) is the main cause of almost 70% of errors in clinical practice. Other signs of ineffective teamwork, such as a lack of shared goals, situational awareness, role clarity, leadership, coordination, and mutual respect, have been associated with poor patient outcomes, for example, hospital-acquired infections, adverse drug events, and increased risk of mortality. Training when used with simulation allows skills to be performed in a consequence-free environment, where mistakes are learning opportunities, allowing the student to receive positive feedback focused on their improvement.

### 7.8 Instructor training

In terms of the effectiveness of simulation, the role of the instructor in facilitating, guiding, and motivating students is still uncertain. There is an unmet need to standardize the education, evaluation, and certification of simulation instructors for the health professions. Despite the lack of research in the area, the observation of some experiences brings important contributions. For example: clinical experience alone is not a prerogative for the effectiveness of a simulation instructor, and simulation instructors and students do not need to be from the same health profession. Many healthcare simulation technology companies offer training courses, once the materials have been purchased, to the staff responsible for handling the equipment. In addition, simulation instructor courses are increasingly available at faculties of education for health professions and trade associations. In terms of the effectiveness of simulation, the role of the instructor in facilitating, guiding, and motivating students is still uncertain. The value of training and recertifying professionals is undeniable. However, the short- and long-term value and usefulness of these educational opportunities are still unknown and lack reliable data in the literature. Several simulation research groups have emerged, linked to medical specialties such as anesthesiology, emergency medicine, internal medicine, obstetrics and gynecology, pediatrics, and surgery. There is no doubt that the technology brought together with simulation can produce substantial educational benefits. However, the effectiveness of this technology requires perseverance and attention to the values and priorities that a healthcare student needs.

## 8 Implementation of the skills laboratory and clinical simulation room at Unicamp

In 2007, during a Congress of Pediatrics in São Paulo, a group of physicians from the Pediatric Emergency Department at Unicamp’s clinical hospital (*Hospital de Clínicas*) after a presentation on the simulation technique, envisioned the implementation of this innovative teaching method at the institution. This technique would allow students to interact more with procedures and clinical cases based on urgent and emergency issues. These dynamics would make up for the deficiency in teaching since, historically, situations requiring an emergency approach in real environments have been decreasing and, on the other hand, the number of students on the medical course was on an increasing scale. The embryo of the current reality was born. At the same time, the University had already started building the Center for Realistic Simulation, in the physical area of the Faculty of Medical Sciences. The Simulation Center was inaugurated in 2009 with a structure comprising an imaging room—equipped with microscopes and computers—arenas, a high-fidelity simulation room and a care simulation room.

The high-fidelity simulation room consists of two simulation rooms and a debriefing room. The rooms contain equipment and materials similar to an adult and pediatric intensive care unit, with an adult high-fidelity simulator, a pediatric simulator, and a childbirth simulator. It also has an audio and video system with five cameras. The care simulation room has a structure similar to a physician’s office with four video cameras and an audio system. In these two rooms, clinical simulation activities are carried out, where students train cognitive and behavioral skills, mainly for the development of clinical reasoning.

In the arenas, the activities accomplished are mainly for the development of technical skills with low and medium fidelity simulators. The structure makes it possible to develop practical stations according to the objective structured clinical examination method. Thus, both in practical activities and in evaluations, participants rotate in time-bound stations so that they can practice technical skills or even practice communication and other non-technical skills.

## 9 Medical simulation: a new didactic strategy for emergency teaching in pediatrics

### 9.1 Proposal for the implementation of medical simulation activities in pediatrics at Unicamp

The Department of Pediatrics operates from the 1st to the 6th year of the undergraduate course in medicine, with specific teaching activities within the logic of increasing complexity. In the 6th year, it is responsible for a subject (MD-131) whose main objective is the immersion in knowledge about Urgency and Emergency in Pediatrics. Its fields of work are emergency care units and referred urgencies. To this end, it has eight tutors (six assistant physicians and two full-time professors) with training in Emergency Pediatrics and Intensive Care, who guide the practical activities and teach the theoretical classes. These activities are accomplished every 6 weeks, for groups of 12–13 students, continuously, with a total of 8 classes per year. From 2013 onwards, the proposal was to incorporate medical simulation activities into the subject, using the physical infrastructure of the skills laboratory and the medical simulation room. The area for these activities also has an amphitheater, a control room, and a debriefing room. To this end, the subject tutors undertook the “I Basic Course for Simulation Instructors” in November 2010. Once this teaching strategy has been fully implemented in medical undergraduate course and residency program, the intention was to offer this training to pediatricians and non-pediatricians at emergency care units in the municipality of Campinas and the metropolitan region.

### 9.2 Rationale for the proposal

In view of all the data and facts presented so far, it is evident that a new pedagogical instrument in medical education and training, including pediatrics, has been forming over the last three decades. It should be noted that this teaching proposal arrived late in our country, with few institutions making use of this “new” instrument. Likewise, in addition to being a teaching tool, it presents an unexplored and vast field for research in the area, including pediatrics. In terms of research, papers have already been presented at national congresses (Curricular Innovations Symposium 2009 and I São Paulo (State) Congress of Urgencies and Emergencies in Pediatrics 2012) and international congresses (International Pediatric Simulation Symposia and Workshops – IPSSW 2010 and 2011), with the design of the proposal, as well as the submission of an article in a medical education journal.

The proposal involves the following activities:

Teaching activities:

(I) 4th medical year: develop skills in common procedures in Pediatric Emergency Units: venipuncture, cerebrospinal fluid puncture, and chest drainage. Knowledge of critical patient care guidelines, through group discussions and seminars with directed themes.
(II) 5th medical year: develop skills in advanced support procedures in Pediatrics, in 3 different activities: vascular access and intraosseous puncture, airway access, and heart rhythm disorders.
(III) 6th medical year: medical simulation, with scenarios that reproduce emergency room care in Pediatrics. Activity carried out in a simulation room with manikins.
(IV) Medical Residency: develop skills in common procedures in Pediatric Emergency Units: arterial puncture, cerebrospinal fluid puncture, chest drainage, and advanced support procedures in Pediatrics. Medical simulation with scenarios of clinical situations in Urgency and Emergency in Pediatrics, repeated periodically, with at least 4 activities in 2 years of Basic Residency in Pediatrics.

The scenarios proposed in items 3 and 4 include the 4 most common situations in Urgency and Emergency in Pediatrics, namely: acute respiratory failure with evolution to respiratory failure, cardiorespiratory arrest, septic shock, and heart rhythm disturbance (tachyarrhythmia). In each scenario, the evaluators will analyze the team’s performance by filling in a “checklist” containing the skills to be achieved (Table 1).

**Table 1:** Team performance analysis model with a checklist containing the skills to be achieved according to the proposed scenario.

Acute respiratory failure	Yes	Partially
1- Assess ABC	2.0	1.0
2- Identify acute respiratory failure as a priority	1.5	0.75
3- Provide O_2_ under various devices	1.0	0.5
4- Indicate orotracheal intubation	1.5	0.75
5- Pre-oxygenation	0.5	0.25
6- Sedation	0.5	0.25
7- Choice of cannula	1.0	0.5
8- Choice of blade	1.0	0.5
9- Check cannula position	1.0	0.5

Cardiorespiratory arrest	Yes	Partially
1- Identification of cardiopulmonary arrest	2.5	1.25
2- Welcoming the companion	1.0	0.5
3- Airway	1.5	0.75
4- Chest compressions	1.5	0.75
5- Vascular access	1.5	0.75
6- Pharmacological management (epinephrine)	1.0	0.5
7- Pulse/rhythm identification	1.0	0.5

Septic shock	Yes	Partially
1- Identify shock	2.0	1.0
2- Identify sepsis	1.5	0.75
3- Assess ABC	1.5	0.75
4- Best time for orotracheal intubation in shock	1.0	0.5
5- Initiate volume replacement	1.5	0.75
6- Choice of crystalloid solution	1.0	0.5
7- Volume of infusion (20 mL/kg)	0.5	0.25
8- Infusion rate (at least 20′)	0.5	0.25
9- Reassessment of “C” at the end of each expansion	0.5	0.25

Supraventricular tachycardia	Yes	Partially
1- Identify sudden event	1.5	0.75
2- History/monitoring	1.5	0.75
3- Initial O_2_ management/access	1.5	0.75
4- Diagnosis Tachyarrhythmia	1.5	0.75
5- “Mechanical” treatment	1.0	0.5
6- Pharmacological treatment	1.0	0.5
7- Electrical treatment (preparation)	1.0	0.5
8- Electrical treatment (execution)	0.5	0.25
9- Sedation/analgesia	0.5	0.25

A: airway, B: breathing, C: circulation, O_2_: oxygen.

*Research activities*: Includes Master’s and Doctoral students in the health education concentration area of the Graduate Program in Clinical Medicine and the Graduate Course in Child and Adolescent Health at Unicamp. Three research projects were developed using this strategy:

o Use of the realistic simulation strategy for medical students in the incorporation of knowledge in Emergency Pediatrics.
o Evaluation of teamwork using the “Debriefing” activity in medical simulation in Pediatric Emergency situations.
o Impact of the training in Emergency Pediatrics of medical professionals, using the Medical Simulation strategy.

The objective is to extend the publications to medical education journals of international circulation (Table 2). In national publications, the objective would be to disseminate knowledge of this teaching strategy to other medical schools, serving as a model for changing the curriculum.

**Table 2:** Description of the studies accomplished in the simulation environment of the University of Campinas.

Article	Population	Methods	Results	Conclusions
Specian Junior et al. (87)	Fourth-year medical students.	Students answered 40 clinical vignette-type multiple choice questions, split evenly by complexity level (knowledge application and integration). Cognitive workload was assessed via eye-tracking, analyzing fixations and revisitations.	Cognitive workload was higher for high-complexity questions compared to low-complexity questions and for participants who answered incorrectly compared to those who answered correctly.	Eye-tracking is a promising tool for identifying the response process validity of clinical vignette-type multiple choice questions, improving their design and providing feedback to enhance teaching and learning.
Soares et al. (88)	Nurses, nurse’s assistant/technician and physicians and medical residents who work in the surgical center.	Authors’ participatory and observational experiences creating the escape room activity.	The escape room was implemented for training safe surgery protocol. Participants were engaged and reported that they were also able to train non-technical skills while training the protocol. During the training, participants worked together and were able to think critically, while having fun and learning.	The escape room strategy effectively reinforced the Safe Surgery Protocol by highlighting weaknesses and engaging the team in revisiting familiar content innovatively.
Santos et al. (89)	Doctor, nurses, nursing technicians.	Cross-sectional pre- and post-test design to assess knowledge and skill improvements after the training of intensive care and emergency healthcare professionals, regarding protocols implement in coronavirus disease 2019 (COVID-19) patient management.	There was a significant increase in test scores from before the training to after the simulation training.	Simulated training enhanced healthcare professionals’ understanding of the COVID-19 protocol, proving valuable for implementing new protocols.
Silva et al.(90)	Nursing undergraduates.	A randomized clinical trial assigned nursing students to control (CG) and experimental (EG) groups, analyzed before and after receiving lectures or lectures plus simulation.	Pre- and post-intervention scores showed that CG students scored a mean of 11.12 before and 12.91 after the intervention, while EG students scored 10.71 and 12.43, respectively. However, no significant difference was found between the groups.	Clinical simulation effectively developed clinical reasoning in nursing students for wound evaluation and treatment, but did not improve knowledge acquisition compared to lectures alone.
Amorim et al. (91)	Sixth-year medical students.	Cross-sectional, observational, analytical study where students participated in four simulated scenarios. Performance was scored using a checklist, and feedback on the activities was collected through three questionnaires assessing prior knowledge, simulation quality, and the debriefing’s contribution to learning.	Among 102 sixth-year medical students evaluated in 32 scenarios, performance was highest in septic shock and lowest in heart rhythm disorders. Most students agreed that realistic simulation was a positive experience, and that this teaching strategy should be a mandatory part of the medical education curriculum.	Students found realistic simulation with debriefing in pediatric emergencies effective for consolidating knowledge and skills, surpassing traditional methods. Video playback during debriefing had limited impact, and performance highlighted the need to prioritize specific pediatric topics.
Schweller et al. (92)	Internal medicine residents.	Residents participated in four weekly simulation sessions, creating two cases based on challenges from their clinical practice. During these sessions, the supervisor played the resident role and returned as facilitator for the debriefing. Extended debriefings addressed the emotional impact of professional dilemmas.	Residents created complex scenarios combining multiple conflicts or ethical dilemmas, aiming to test physicians’ emotional responses during crises. They expected emotional outbursts but were surprised to witness supervisors resolving conflicts calmly and wisely, leading to significant learning.	Creating the cases triggered essential reflections that brought subconscious issues and residents felt confident to share their challenges during debriefings. This innovative approach introduces a new dimension to simulation training, addressing trainees’ personal needs.
Silva and Oliveira-Kumakura (93)	Nursing undergraduates.	Experience report of the creation of two clinical scenarios aimed to improve students’ clinical reasoning in assessing adult and elderly patients. Students completed pre-simulation activities and discussed cases. The simulations focused on wound care, followed by debriefings on emotions and clinical analysis.	The scenarios simulated nursing care using role play and moulage, enabling the evaluation and discussion of wound treatment. The debriefing reflections were crucial for linking theory to practice, contributing to the students’ satisfaction with the activity.	Using clinical simulation scenarios to teach students favored clinical reasoning and decision-making in the evaluation and treatment of wounds.
SciELO Brasil (94)	Nursing undergraduates.	Experience report on the topic, “Nursing care for the patient with burns.” Strategies during this course involved theoretical lecture, discussion of clinical cases, use of a virtual environment, and practice in a simulated environment.	The students reported satisfaction with the tools used.	Incorporating diverse active teaching strategies, such as clinical simulations, e-learning, dialogue-based classes, and case studies, is essential for undergraduate nursing education on burn care.
Schweller et al. (95)	First-year medical students.	Pretest-posttest study evaluated the empathy levels (using Jefferson Scale of Physician Empathy; JSPE) of medical students before and after a four-month didactic intervention. The course focused on values and virtues related to medical professional identity, incorporating real-world activities such as patient and physician interviews, hospital visits, and simulated consultation discussions.	The mean pretest JSPE score was 117.9 and increased to 121.3 after the intervention. The difference was significant. The observed increase was greater among students with lower initial JSPE scores.	Empathy is key to a successful physician-patient relationship and reflects other virtues of a good physician. This study suggests that early curricular interventions can enhance medical students’ empathy and positively influence their professional identity, even when empathy is not the central focus.
Costa et al. (96)	Medical students.	Data from three studies of 3,069 medical students across five countries were analyzed to explore empathy using the JSPE and Davis’s Interpersonal Reactivity Index (IRI). Surveys assessed empathy, psychological well-being, and attitudes toward end-of-life care.	Correlation analysis showed weak but significant relationships between IRI and JSE-S scores. Regression models found IRI subscales to be weak predictors, explaining 9–15.3% of variance in JSE-S subscales and 19.5% in total scores.	Factor analysis confirmed the IRI and JSE-S structures, linking their subscales to empathy’s affective and cognitive dimensions. Findings across five countries showed these tools measure distinct concepts: the IRI reflects dispositional empathy, while the JSE-S focuses on context-specific empathy.
de Araujo Guerra Grangeia et al. (97)	Sixth-year medical students.	Weekly “Virtual Rounds” simulated real emergency rounds, supplemented by activities like Extreme Decisions, Emergency Quiz, and electrocardiogram challenges. Participation was correlated with academic performance, and surveys assessed student opinions, cognitive load, and motivation.	Students averaged 1,965 page views and 72 h online, with course access extending beyond the two-month Clinical Emergency rotation to an average of 5.3 months. “Virtual Rounds” was the most accessed activity, showing a positive correlation between platform usage and final grades. Over 90% of students reported improved clinical reasoning and readiness for emergency care. Cognitive load scores were 4.79 ± 2.2 for Virtual Rounds and 5.56 ± 1.96 for real rounds.	An online course based on real patients and emergency care narratives can effectively provide a theoretical foundation in Emergency Medicine while building student confidence to apply learning in the Emergency Room.
Schweller et al. (98)	Fourth and sixth-year medical students.	Students completed the JSPE before and after simulated consultations with standardized patients, followed by debriefings addressing patient emotions (fear, guilt, anger, and abandonment), doctor-patient dynamics, and related topics.	The simulation activity increased the empathy scores of the fourth-year students (from 115.8 to 121.1) and of the sixth-year students (from 117.1 to 123.5).	Findings suggest that simulating medical consultations with standardized patients can enhance medical students’ empathy and provided a platform to discuss the doctor-patient relationship, the hidden curriculum, negative role models, and emotional experiences.

*Extension activities*: Promote training courses in Pediatric Procedures and Emergencies for professionals from the municipal and regional health network. The objective is the training of teams on the model of the short courses already offered by associations that use the principles of Basic and Advanced Support in Pediatrics.

So far, the activities proposed for the 6th year of medicine and the 1st year general pediatrics residents have been carried out. The current work proposal is as follows:

#### 9.2.1 MD-131—comprehensive child health care

*Summary*: Develop knowledge, skills, and attitudes in relation to newborn, child, and adolescent health care. In-service activities in Pediatric Emergency Units of secondary and tertiary complexity and practice of hospitalization in General Ward in Pediatrics and Neonatology Unit of secondary complexity. Theoretical and practical activities on the most prevalent pediatric diseases. Ethics.

Specificity related to the discipline in the simulation environment:

o Application of clinical simulation scenarios that reproduce the care of children with shock, traumatic brain injury, cardiorespiratory arrest, and respiratory failure. A high-fidelity pediatric simulator is used, as well as audio and video resources.
o Practical activity for 6th year students, where training in technical skills such as orotracheal intubation, cardiopulmonary resuscitation, and intraosseous injection is accomplished. A low-fidelity simulator is used for these activities.

#### 9.2.2 MD-136—emergency

*Summary*: To develop competencies for integrated care in the areas of Emergency and Adult Urgent Medical Care: syndromic diagnosis, first care measures, and clinical and surgical follow-up. Training in the physician-patient relationship, communication skills, clinical ethics, and palliative care.

Specificity related to the discipline in the simulation environment:

o Applying clinical simulation scenarios that reproduce the care of patients with acute respiratory failure, shock, sepsis, tachyarrhythmias, and bradyarrhythmias. The simulation room is used with an adult high-fidelity simulator, as well as audio and video resources.
o Application of clinical simulation scenarios, using simulated patients, which reproduce real care situations in order to develop communication and empathy skills. The consultation simulation room and audio and video resources are used.

#### 9.2.3 MD-138—trauma surgery

*Summary*: Practical activities in the referenced emergency unit and in the ward, operating room, intensive care unit, outpatient clinic and mobile emergency care service, in surgical clinic and surgical emergencies, monitoring critically ill patients, participating in diagnosis and treatment. Indicating and interpreting subsidiary tests, conducting pre- and post-operative care, recognizing the main post-operative complications. Ethics.

Specificity related to the discipline in the simulation environment:

o Application of clinical simulation scenarios involving initial care for polytraumatized patients, rapid intubation sequence, and shock. The simulation room is used with a high-fidelity adult simulator, as well as audio and video resources.
o The practical test is carried out with 6th year students, using low-fidelity manikins for the procedure stations and medium-fidelity simulators for the clinical case service stations.

#### 9.2.4 MD-943—comprehensive adult health care I

*Summary*: Practical training in the areas of clinical and surgical cardiology, infectious diseases, sexually transmitted diseases, dermatology with an emphasis on leprosy, general oncology, and immunology. Clinical, outpatient and follow-up care for hospitalized patients are carried out. Familiarization with prevalent and important diseases in each area, with guidance on clinical history, physical examination, propaedeutics, and therapy.

Specificity related to the discipline in the simulation environment:

o Application of clinical simulation scenarios that reproduce the care of patients with pulmonary thromboembolism, aortic dissection, cardiorespiratory arrest, and arrhythmias. The simulation room is used with a high-fidelity adult simulator, as well as audio and video resources.

#### 9.2.5 MD-344—skills Laboratory I

*Summary*: Identification of victims in critical situations. Traumatized patients. Basic resuscitation measures, life support, and first aid. Principles of pre-hospital care.

Specificity related to the discipline in the simulation environment:

o Practical activities developed with low-fidelity simulators that allow students to practice basic life support, cardiopulmonary resuscitation, patient immobilization, and victim transport.

#### 9.2.6 MD-758—comprehensive adult health care II

*Summary*: Supervised outpatient care in Basic Health Units, covering comprehensive child, adolescent and adult health care, including the specificities of women’s health. Clinical, diagnostic and therapeutic approach to the most prevalent diseases and symptoms in the different age groups. Collective Health, Occupational Health, Environmental Health, Clinical Pathology, Radiology, Mental Health, Family Health, and Ethics.

Specificity related to the discipline in the simulation environment:

o Clinical simulation scenarios for 4th year students involving the care of patients with diabetes mellitus. It uses the consultation simulation room, audio and video resources.

#### 9.2.7 MD-643—semiology and propaedeutics

*Summary*: Theoretical-practical activities in physician semiology, pediatrics, and tocogynecology, with participation in the daily care of patients in the hospital complex.

Specificity related to the discipline in the simulation environment:

o Practical activities developed with low-fidelity simulators that allow students to develop technical skills for gynecological and obstetric examination.
o Activities with the high-fidelity simulator to demonstrate the mechanism of childbirth.

#### 9.2.8 Other subjects, courses and programs involved

*Interprofessional simulation course*: Use of 7 clinical simulation scenarios for patient care in a Basic Health Unit to develop non-technical skills in students, such as communication, teamwork, leadership, and decision-making with a focus on patient safety. These scenarios use the structure of simulation rooms with all their resources (high-fidelity simulator, video cameras, and audio equipment).

*FN-466—principles of dysphagia*: This course introduces speech therapy students to clinical simulation activities. A dysphagia patient care scenario is applied, using the adult high-fidelity simulator and the resources of the simulation room.

*Multiprofessional residency*: While still under development, the clinical simulation scenario for the multiprofessional residency program will involve nursing, speech therapy, physiotherapy, and pharmacy. The simulation will use the adult high-fidelity simulator and audio and video resources in the simulation room.

*Pediatrics residency*: Application of clinical simulation scenarios for Pediatrics residents involving the care of children with shock, traumatic brain injury, cardiorespiratory arrest and respiratory failure. A high-fidelity pediatric simulator is used, as well as audio and video resources in the simulation room.

*Tocogynecology residents*: A childbirth care course is offered to Tocogynecology residents, and one of the activities in this course is clinical simulation. Simulation scenarios are used to deal with obstetric emergencies. A high-fidelity childbirth simulator and audio and video resources are used.

## 10 Conclusion

Working in an emergency requires that, as well as doing the right thing, the turnaround time be as short as possible. This requires professionals to have technical skills, excellent communication, leadership and, above all, team management. In addition to defining the diagnosis and the appropriate course of action, we are also responsible for patient safety. Therefore, anticipating risk situations that may occur in routine healthcare operations is essential in simulation training, as a strategy to support organizations in their quest for high reliability. By recognizing and betting on the potential of Simulation, both for improving educational practices in health teaching and for popularizing and widely using it as a method, it is expected that, in the coming years, studies and the dissemination of successful experiences can contribute to reducing the uncertainties and limitations related to the effectiveness of these technologies.
